# Viral Spectrum of Herpetic Keratitis: A 15-Year Retrospective Analysis from Switzerland

**DOI:** 10.3390/microorganisms14020268

**Published:** 2026-01-23

**Authors:** Muntadher Al Karam, Sadiq Said, Anahita Bajka, Irene Voellmy, Michael Huber, Sandrine A. Zweifel, Daniel Barthelmes, Frank Blaser

**Affiliations:** 1 Department of Ophthalmology, University Hospital Zurich, University of Zurich, 8091 Zurich, Switzerland; muntadher.alkaram@usz.ch (M.A.K.);; 2 Augenklinik Wettingen, 5430 Wettingen, Switzerland; 3 Talacker Augenzentrum Zürich (TAZZ), 8001 Zurich, Switzerland; 4 Institute of Medical Virology, University of Zurich, 8057 Zurich, Switzerland

**Keywords:** herpetic keratitis, viral keratitis, infectious keratitis, epidemiology, incidence

## Abstract

To evaluate the epidemiology of herpetic keratitis over a 15-year period at a tertiary care center in Switzerland, focusing on the relative incidence of herpes simplex virus (HSV)-1, HSV-2, and varicella zoster virus (VZV), gender distribution, and co-infections, we conducted a retrospective single-center analysis of polymerase chain reaction (PCR) assays from corneal and conjunctival scrapings of suspected herpetic keratitis at a tertiary referral hospital. Patient demographics, viral spectra, and microbiological co-infections were assessed. Between 2010 and 2025, we identified 9954 PCR assays from 2892 patients, with 482 samples testing positive for herpesvirus. HSV-1 was the most frequent pathogen (328 of 3358, 9.8%), followed by VZV (143 of 3112, 4.6%), HSV-2 (9 of 3290, 0.27%), and CMV (2 of 194, 1.0%). Triplet testing (simultaneous HSV-1, HSV-2, and VZV-PCR) enabled direct comparisons of relative incidence rates. We found 2913 triplet testing results, with a relative distribution in positive results of 65.4% for HSV-1, 32.5% for VZV, and 2.1% for HSV-2. HSV-1 keratitis had a statistically significant higher incidence in men (58.9%, *p* = 0.0044), while no sex difference was detected for VZV (47.9%, *p* = 0.6683), HSV-2 (33.3%, *p* = 0.5078), or CMV (100%, *p* = 0.500). Bilateral infections were present in two patients, and co-infections were detected as follows: 8 cases of HSV-1/VZV co-detection, 3 cases of Acanthamoeba, and 15 of fungi. HSV-1 was the overwhelmingly dominant cause of herpetic keratitis at our institution, occurring more than twice as frequently as VZV and vastly outnumbering HSV-2. The statistically significant higher incidence in men in HSV-1 keratitis suggests possible biological or sociodemographic influences, whereas co-infections highlight the complexity of corneal pathology in a referral setting. These findings underscore the importance of multiplex PCR testing for accurate pathogen detection and provide insights into the epidemiologic landscape of herpetic keratitis.

## 1. Introduction

Herpetic corneal infections constitute a leading cause of infectious keratitis and corneal blindness worldwide [1,2]. The primary causative viruses include herpes simplex virus types 1 and 2 (HSV-1 and HSV-2), varicella zoster virus (VZV), and cytomegalovirus (CMV). Among these, HSV keratitis, predominantly due to HSV-1, is the most prevalent, and is recognized as the most common cause of infectious corneal blindness in developed countries [3]. Approximately one in five individuals with ocular HSV infection develops stromal herpes simplex keratitis, carrying a high risk of visual impairment [4]. Globally, the incidence of herpes keratitis is estimated at approximately 24 per 100,000 people per year, resulting in roughly 1.7 million new infections annually and causing substantial ocular morbidity, including vision loss due to recurrent disease and corneal scarring [5,6,7].

**Figure 1:**
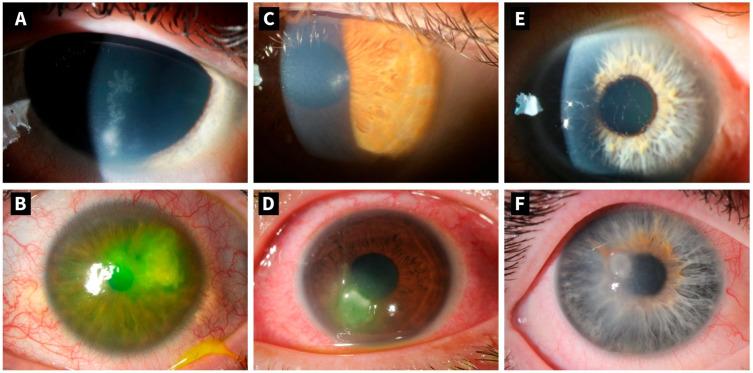
Slit lamp photography of different clinical presentation of herpetic keratitis of patients from the department of ophthalmology of the University Hospital Zurich, Switzerland: (**A**) epithelial keratitis of HSV-1 with typical dendritic lesion with terminal bulbs; (**B**) geographic keratitis of HSV-1 with stromal ulceration with fluorescein dye; (**C**) epithelial keratitis of HSV-2; (**D**) ulclerative keratitis of HSV-2 with fluorescein dye; (**E**) epithelial keratitis of VZV with pseudodendrites with no central ulceration and no terminal bulbs; (**F**) ulcerative keratitis of VZV with pseudodendrites at the margin of the lesion.

Diagnostic methods for herpes keratitis include clinical examination using slit-lamp biomicroscopy, polymerase chain reaction (PCR) assays, viral culture, immunofluorescence staining, and confocal microscopy [8]. PCR testing is the gold standard of invasive procedures due to its high sensitivity and specificity, enabling rapid and precise identification of the causative virus [9]. Moreover, it is critical to distinguish herpes keratitis from other infectious causes, such as bacteria, fungi, or parasites, especially acanthamoeba. Herpes keratitis may be grouped clinically into epithelial, stromal, endothelial, or neurotrophic keratitis. The epithelial affection typically manifests with dendritic lesions visible through slit-lamp biomicroscopy. In contrast, stromal keratitis is characterized by corneal stromal inflammation, stromal opacities, edema or ulceration, and potential subsequent stromal scarring [9,10]. Figure 1 shows clinical manifestations of herpetic keratitis caused by HSV-1, HSV-2, and VZV. Endothelial herpetic keratitis presents with disciform stromal edema, keratic precipitates (usually localized in the area of the edema), anterior chamber reaction, and usually absent epithelial defect (in contrast to epithelial keratitis). As a varicella-zoster of all herpetic corneal infections, neurotrophic keratitis may occur, resulting in impaired corneal sensation and persistent epithelial defects [11,12].

The overlapping clinical features of herpes virus keratitis make it challenging to distinguish among the different entities solely on clinical examination. HSV epithelial keratitis typically presents true dendrites demonstrating central fluorescein uptake, whereas VZV pseudo-dendrites lack central ulceration and therefore do not stain centrally [13]. CMV keratitis is most often seen in immunocompromised patients or following organ transplantation [14]. Definitive diagnosis, however, relies on pathogen detection through corneal scrapings or anterior chamber paracentesis [14].

Despite advances in diagnostics and therapy, herpes keratitis remains a significant challenge owing to its recurrent nature, diverse clinical manifestations, and risk of severe visual morbidity. Our tertiary care center in Switzerland functions as a national referral center for atypical or severe keratitis, including herpes-related cases. This retrospective study assesses the incidence and patient characteristics of PCR-confirmed herpes keratitis at our institution and reviews the current literature.

## 2. Materials and Methods

This is an investigator-initiated, retrospective, single-center study conducted at the University Hospital Zurich in Switzerland. We identified patients diagnosed with PCR-positive herpes keratitis between January 2010 and March 2025. The leading ethics committee in Zurich waived our study protocol as it does not fall within the scope of the Human Research Act (BASEC number 2023-01146). Nevertheless, we handled all data according to Good Clinical Practice guidelines.

### 2.1. Data Collection

We reviewed our laboratory information system of patients regarding PCR-positive corneal or conjunctival scraping results for HSV-1, HSV-2, VZV, and CMV. We extracted the patients’ birth dates, gender, and date of positive results, inferring the patients’ age at the time of test positivity. To explore co-testing patterns and interrelationships, we identified all “triplet” tests in which a single specimen was assayed simultaneously for HSV-1, HSV-2 and VZV. Within this triplet cohort, we compared detection rates across the three viruses. Further, we investigated for Acanthamoeba and fungal co-infections, considering PCR and culture results. Bacterial co-infections were not assessed in this study, as they are presumably common, culture positivity in corneal scrapings is often due to probe contamination, and all infectious keratitis patients at our clinic receive empirical topical antibiotics before a definitive diagnosis. However, we routinely include bacterial investigations in all corneal scrapings in unclear, presumed-infectious keratitis.

### 2.2. Detection of CMV, HSV-1, HSV-2, and VZV

Sample preparation and DNA extraction: For microbiological diagnostics, corneal scrapings were used as the primary sampling method; conjunctival swabs were obtained only in exceptional cases, such as in pediatric patients or when patient cooperation was insufficient to safely perform corneal scraping. Corneal and conjunctival swabs were placed in 2 mL viral transport medium (VTM) immediately after collection. Before sample processing, specimens were vortexed, swabs removed, and the remaining VTM centrifuged at 2000 relative centrifugal force (rcf) for 10 min. DNA was extracted using easyMAG and EMAG^®^ nucleic acid extraction systems (bioMérieux, Marcy l’Etoile, France) according to the manufacturer’s protocol from 200 microliter (μL) supernatant and concentrated in 110 elution buffer (extraction buffer 3). From 28 August 2024, nucleic acid was extracted from 600, 400, or 300 μL supernatant, depending on the number of additional analyses requested, and concentrated in 60, 40, or 30 μL elution buffer (extraction buffer 3), respectively. In addition, all specimens were spiked with a standardized amount of Phocid herpes virus type 1 (PhHV) as PCR process control before extraction (European virus archive EVAG, Marseille, France, https://www.european-virus-archive.com) [15].

DNA amplification: DNA PCR amplifications were conducted following in-house real-time PCR protocols. All primers and Taqman probes were synthesized by Microsynth (Microsynth AG, Balgach, Switzerland). The PCR tests used in this study are validated diagnostic tests with high sensitivity and specificity. The assays are specific for the individual Herpesvirus species within the Simplexvirus genus or the Orthoherpesviridae family [15,16,17,18,19]. Sequences of all primers and probes are listed in Table 1.

Until 28 August 2024, a single-plex PCR process control using GAPDH (Homo sapiens glyceraldehyde-3-phosphate dehydrogenase) was run separately for HSV-1, HSV-2, and VZV PCR tests. For CMV, the GAPDH process control was run as a duplex assay with GAPDH primers and probe incorporated in the CMV-Mastermix. Each viral target assay was performed in duplicate to increase detection sensitivity. Mastermixes for HSV-1, HSV-2, VZV, and GAPDH PCR process controls were composed of 900 nM of each respective target primer and 200 nM of each respective target probe in 25 μL of TaqMan Universal MMIX II (Applied Biosystems by Thermo Fisher Scientific, Waltham, MA, USA) and 19 μL nuclease-free water (AppliChem GmbH, Darmstadt, Germany). CMV Mastermixes contained 900 nM of CMV primers, 100 nM of GAPDH primers, and 200 nM of CMV and GAPDH probe in 25 μL of TaqMan Universal MMIX II (Applied Biosystems) and 18.8 μL nuclease-free water (AppliChem GmbH) or, from 5 May 2023, 17.9 μL nuclease-free water. All RT-PCR amplifications were conducted in a total volume of 50 μL, containing 45 μL of Mastermix and 5 μL of eluted DNA.

From 28 August 2024, GAPDH process controls were replaced by the amplification of phocid herpes virus (PhHV) glycoprotein B in combination with spiking all clinical specimens with a standardized amount of PhHV. Each PCR assay was run in a duplex protocol, also containing primers and TaqMan probe for PhHV detection. Mastermixes from 28 August 2024 thus contained 780 nM of each respective diagnostic target primer, 50 nM of PhHV forward primer, 200 nM of PhHV reverse primer, 200 nM of respective diagnostic target probe and 100 nM of PhHV probe in 21.7 μL of TaqMan Universal MMIX (Applied Biosystems) and 17.3 μL nuclease-free water for HSV-1, HSV-2, and VZV or 16.5 μL nuclease-free water for CMV (AppliChem GmbH). All RT-PCR amplifications were conducted in a total volume of 50 μL, containing 40 μL of Mastermix and 10 μL of eluted DNA modifications of the new protocol (i.e., DNA extraction protocol leading to a 10-fold concentration of eluted DNA and doubling the volume of eluted DNA added to the PCR reaction mix) resulted in a 10-fold increase in sensitivity with potentially higher target detection rates. Thus, assay duplicates were eliminated. DNA amplifications were conducted in a QuantStudio™ 3 Real-Time PCR System (Applied Biosystems) under the following conditions: 2 min at 50 °C, 10 min at 95 °C and 50 cycles of 15 s at 95 °C, and 1 min at 60 °C. In each run, negative and positive controls were included. Elution buffer (NUCLISENS^®^ easyMAG^®^ Extraction Buffer 3, bioMérieux) was used as negative control and target-specific positive controls were all obtained from ATCC [ATCC, Manassas, VA, USA] as follows: CMV: purified viral DNA, ATCC VR-538, strain AD-169; HSV-1: purified viral DNA, ATCC VR-539, strain MacIntyre; HSV-2: purified viral DNA, ATCC VR-734, strain G; VZV: purified viral DNA, ATCC VR-586, strain Ellen. Signals reaching cycle thresholds of <38 were interpreted as positive; tests raising signals after ≥38 cycles were repeated and reported as positive if a signal was also detected in the repeated test. If assays were run in duplicates, tests were interpreted as positive, if at least one test result was positive. Signals of GAPDH or PhHV process controls were required before 33 cycles, i.e., the reaction showed no inhibition or reduction of DNA during extraction and PCR [16,17,18,19,20].

### 2.3. Statistical Analyses

In descriptive statistics, we present means with standard deviation and medians with interquartile ranges (IQR), or minimum to maximum values for continuous data and numbers and percentages for categorical data. To assess potential gender predominance, we tested whether the observed proportion of male positive results for each virus differed significantly from a neutral distribution of 50%. For each pathogen (HSV-1, HSV-2, VZV, and CMV), we performed a two-sided exact binomial test under the null hypothesis that the probability of a positive result being male was 0.5. To account for multiple comparisons across the four pathogens, we applied a Bonferroni correction, adjusting the significance threshold from α = 0.05 to α = 0.0125 (0.05 divided by 4). We considered a *p*-value statistically significant only if it was below the corrected threshold and the corresponding 95% confidence interval (CI) for the male proportion did not include 0.5. All statistical analyses were conducted using R version 4.2.1 (R Foundation for Statistical Computing, Vienna, Austria).

## 3. Results

From January 2010 to March 2025, we identified a total of 9954 herpesvirus PCR test results performed on corneal and conjunctival scrapings. Demographic information was available for 8693 individual test entries, corresponding to 2892 patients. Hence, all the following demographics were only conducted on the latter dataset. The median (IQR; range) patient age was 48.0 (31.7–66.3; 0–97) years, with a quasi-balanced gender distribution (49.7% female). In total, 3358 PCR tests were targeted for HSV-1, 3290 for HSV-2, 3112 for VZV, and 194 for CMV. Among these, 328 (9.8%) were positive for HSV-1, 9 (0.3%) for HSV-2, 143 (4.6%) for VZV, and 2 (1.0%) for CMV. Table 2 summarizes the patient demographics stratified by virus type.

We identified 2913 corneal-scraping specimens tested in triplets (i.e., simultaneous testing for HSV-1, HSV-2, and VZV), enabling direct comparisons of detection rates across these three viruses. Among the 419 positive results obtained within the triplet cohort, 274 (65.4%) were positive for HSV-1, 136 (32.5%) for VZV, and 9 (2.1%) for HSV-2. Table 3 displays detailed results of the triplet analyses. For each pathogen, we tested whether the proportion of male patients among positive cases differed from an equal distribution between males and females. Applying a two-sided exact binomial test, we found that 161 of 274 HSV-1 positive cases were male, corresponding to a male proportion of 58.9% (95% CI: 52.7–64.7%), with a *p*-value of 0.0044, indicating a statistically significantly higher incidence in men. For HSV-2, 3 of 9 positive cases were male (33.3%, 95% CI: 7.5–70.1%; *p* = 0.5078), for VZV, 65 of 136 were male (47.9%, 95% CI: 39.2–56.5%; *p* = 0.6683), and for CMV, 2 of 2 were male (100%, 95% CI: 15.8–100%; *p* = 0.5000), none of which differed significantly from equal distribution.

**Figure 2:**
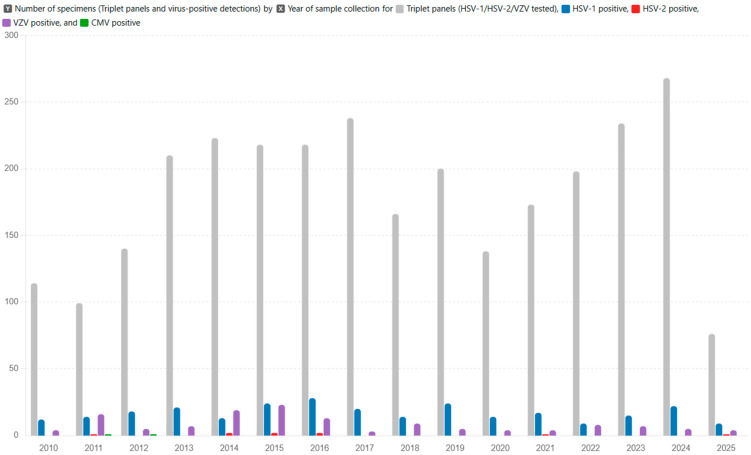
Annual number of triplet test panels and virus detections conducted from January 2010 until March 2025 at the University Hospital Zurich, Switzerland from Table 4 displayed in a diagram.

The use of multiplex PCR testing increased across the study period, reflecting the broader implementation of standardized triplet panels. In 2010, we performed 114 triplet assays, with the number of assays increasing yearly to a peak of 268 in 2024. Notably, there were two transient declines: from 238 assays in 2017 to 166 in 2018, and from 200 in 2019 to 138 in 2020. Despite the overall growth in testing volume, annual positivity rates remained relatively stable (HSV-1: 8–11%; VZV: 3–6%), whereas HSV-2 detections remained rare, with only nine positive triplet assays identified in six separate years (2011, 2014–2016, 2021, and 2025). Table 4 and Figure 2 provide a detailed analysis of the temporal triplet testing patterns.

Demographically, the triplet cohort closely mirrored the overall tested population, with no significant differences in age or gender proportions compared to the full assay set. HSV-2 detections remained rare, with nine unique patients tested positive for HSV-2 keratitis; one patient had two positive results within 10 days, yielding a total of 10 positive assays. The median (IQR) age of HSV-2 positive patients was 57.2 (46.7–66.4) years, and 66.7% were female. Furthermore, we identified eight cases of simultaneous HSV-1 and VZV co-infections, of whom two (25.0%) were female, ranging in age from 16 to 70 years. Clinical documentation reported six epithelial presentations and two corneal ulcers. In addition, two patients presented with simultaneous bilateral viral detections: a 29-year-old male with bilateral HSV-1 infection and a 74-year-old male with bilateral VZV infection.

Regarding other microbiological co-infections, two (0.6%) patients with HSV-1 and one (0.7%) with VZV-positivity had concurrent Acanthamoeba infection. Neither of the HSV-1-positive cases wore contact lenses, whereas the VZV-positive case wore soft daily contact lenses. Furthermore, 15 (3.2%) patients showed positive fungal detections. Of these, 11 (73.3%) were positive for HSV-1, none were positive for HSV-2, and 4 (26.7%) were positive for VZV. Regarding contact lens use, 8 of 11 (72.7%) HSV-1-positive and 3 of 4 (75.0%) VZV-positive patients used contact lenses.

## 4. Discussion

This retrospective study investigated PCR test results regarding possible herpetic keratitis at our tertiary care center in Switzerland. Between January 2010 and March 2025, we identified 9954 PCR tests, obtained from 2892 patients. Over the 15-year interval, the laboratory’s adoption of multiplex triplet panels (HSV-1, HSV-2, VZV) increased markedly—from 114 triplet panels in 2010 to a peak of 268 in 2024—reflecting broader clinical uptake of PCR diagnostics for suspected viral keratitis. These trends underscore the increasing reliance on comprehensive PCR diagnostics in cases of suspected viral keratitis. We found 9.8% and 4.6% positivity for HSV-1 and VZV, respectively, whereby HSV-2 and CMV occurred much less frequently. Despite this expansion in testing throughput, the annual proportion of positive triplet results for HSV-1 and VZV remained relatively constant (HSV-1 around 8–11%; VZV around 3–6%), which supports a stable clinical threshold for testing and the epidemiological dominance of these viruses in corneal disease.

HSV-1 was the overwhelmingly predominant pathogen in our dataset, which aligns with global epidemiology. Worldwide, HSV-1 is the leading cause of herpetic keratitis, with pooled incidence estimates of 24 per 100,000 people per year, most cases affecting the corneal epithelium [3,20,21]. Its predominance is attributable to the high prevalence of latent HSV-1 infection within the trigeminal ganglion and the lifelong risk of recurrent corneal reactivation. VZV was the second-most frequent pathogen in our cohort, which is also consistent with international data: although VZV accounts for only a minority of herpetic keratitis cases (3–12%), it remains an important cause of ocular morbidity due to its association with herpes zoster ophthalmicus [14]. By contrast, HSV-2 was rarely detected (<1%), aligning with previous European and global studies [22,23,24,25]. HSV-2 keratitis most often occurs through neonatal transmission or in immunocompromised patients, and is therefore uncommon in otherwise healthy adults [20,22,25]. Although cases of CMV epitheliitis and CMV stromal keratitis have been reported, CMV endotheliitis is the most common manifestation of corneal infection [26].

In our tertiary care center, we routinely perform multiplex PCR triplet testing, as the clinical presentation of herpetic keratitis does not allow reliable differentiation of the causative virus. In addition, initial therapy differs: current consensus recommends valaciclovir 500 mg three times daily for HSV-1 stromal keratitis, whereas VZV stromal keratitis requires 1 g three times daily [11,12].

Triplet testing in our cohort enabled a direct comparison of the relative incidence rates, with HSV-1 identified in 65.4% of positive assays, VZV in 32.5%, and HSV-2 in 2.2%. Previous studies limited to corneal specimens reported substantially higher proportions of HSV-1 (89%) compared to VZV (11%) [27]. By contrast, investigations including a broader range of ocular samples—such as conjunctival swabs, corneal scrapings, aqueous humour, and vitreous samples—demonstrated a more balanced distribution, with VZV slightly exceeding HSV-1 (12.3% versus 11.7%) [22]. These differences suggest potential tissue tropism: HSV-1 appears to have a predilection for the corneal epithelium and stroma, whereas VZV may more frequently involve multiple ocular structures beyond the cornea. The distribution in our study likely reflects the inclusion of both corneal and conjunctival scrapings, which may have increased the relative detection of VZV while proportionally reducing the apparent dominance of HSV-1 compared to studies analyzing only corneal tissue.

In this study, we observed two transient reductions in triplet testing throughout the observation period. The first decline occurred between 2017 and 2018 (from 238 to 166 triplets) and the second between 2019 and 2020 (from 200 to 138 triplets). The latter coincides with the onset of the COVID-19 pandemic and is plausibly explained by reduced outpatient activity and fewer elective procedures, resulting in fewer samplings. In contrast, the 2018 decline is less clearly attributable to a single external factor and may reflect local workflow adjustments or changes in referral patterns. These year-to-year perturbations highlight the importance of interpreting temporal trends in the context of operational and public health influences, rather than attributing them solely to epidemiologic fluctuations.

Identifying co-infections in patients with herpes keratitis is of crucial clinical importance as they may influence management and final visual outcomes. In our study, we identified cases of Acanthamoeba or fungal co-infection in patients with PCR-confirmed herpes keratitis, consistent with prior reports describing the coexistence or clinicopathologic overlap among Acanthamoeba, fungal, and herpetic keratitis [28,29]. The coexistence of amoeba and herpes may be due to a chance of dual infection or to a symbiosis in which one pathogen masks the other. Overall, contact lens use and ocular trauma remain the primary risk factors for infectious keratitis, which may explain the potential for co-infections [30]. These results emphasize the importance of comprehensive microbiological evaluations in atypical keratitis cases.

We found a statistically significant higher incidence in men in HSV-1-associated keratitis, a gender-associated observation that is difficult to explain and highlights an existing research gap regarding the underlying pathophysiology. The possible reasons for sex-related differences in the incidence of herpetic keratitis are multifactorial and remain incompletely understood. While male predominance has been reported in several studies, the extent and statistical significance of this finding vary across populations and study designs [31,32]. Conversely, not all studies show this pattern, and some pediatric cohorts have even reported a predominance of female HSV-1 infections [33]. Biological factors, such as sex hormone differences and differences in immune response, may contribute. Animal models have shown that male mice can have higher mortality and more severe disease following HSV-1 ocular infection, potentially due to androgen effects and gender-specific modulation of interferon pathways [34,35]. However, such findings have not been consistently replicated in human populations. Sociodemographic and behavioral factors may also play a role. For example, differences in occupational exposure, healthcare-seeking behavior, and risk of corneal trauma could influence gender distribution in certain cohorts [36,37]. Geographic and cultural factors, as well as comorbidities such as diabetes and immunosuppression, may further modulate risk but do not reveal a clear gender predilection [33,36,38]. Taken together, these findings underscore that the true mechanisms driving gender differences in HSV-1 keratitis remain poorly defined and warrant further investigation.

This study’s key strengths include a 15-year observation period and a large data sample of nearly 10,000 PCR assays. Furthermore, the standardized triplet testing of HSV-1, HSV-2, and VZV on the same specimen, along with transparent laboratory methods and internal controls, enables robust within-sample comparisons and temporal descriptions. However, this study has several limitations. First, the retrospective design is inherently prone to selection bias and missing data. The single-center design may introduce referral and sampling biases, potentially leading our study cohort to not accurately reflect other populations and testing practices. Further, incomplete demographic data may affect the accuracy of incidence estimates. Moreover, the mid-study laboratory changes complicate longitudinal comparisons of positivity rates. Last, the rarity of HSV-2 and CMV limits the precision of incidence for those subgroups.

## 5. Conclusions

To conclude, over a 15-year period at our Swiss tertiary care center, HSV-1 emerged as the most common cause of PCR-confirmed herpetic keratitis. This was followed by VZV, whereas HSV-2 and CMV were rarely detected. We advocate for triplet testing panels not only for a more complete and efficient laboratory assessment, but also for epidemiologic monitoring reasons that allow for investigating relative prevalence data. The statistically significant higher incidence of HSV-1 in men underscores a research gap in understanding the sex-specific pathophysiology of herpetic keratitis, emphasizing the need for future biological and sociodemographic studies to investigate the underlying mechanisms.

## Figures and Tables

**Table 1:** Target primers and probes used during the testing period.

Primer/Probe	In Use From	Sequence	Amplicon Region, Length
CMV forward	Before 2010	5′-GCC CAA GAC ATC ACC CAT G-3′	Glycoprotein B, UL55, 62 bp [17]
CMV forward modified 1A6G	From 5 May 2023	5′-ACC CAG GAC ATC ACC CAT G-3′ (allowing detection of additional strains)	
CMV reverse	Before 2010	5′-CCA TTC TCT CGG CCA TTT ACA-3′	
CMV reverse modified 17C	From 5 May 2023	5′-CCA TTC TCT CGG CCA TCT ACA-3′ (allowing detection of additional strains)	
CMV probe	Before 2010	5′-FAM-CAA ACC GAT TGC CGC GCG TTT-TAMRA-3′	
HSV-1 forward	Before 2010	5′-CTG TTC TCG TTC CTC ACT GCC T-3′	Glycoprotein G, ORF 4 US fragment, 81 bp modified from [18]
HSV-1 reverse	Before 2010	5′-CAA-AAA-CGA-TAA-GGT-GTG-GAT-GAC-3′	
HSV-1 probe	Before 2010	5′-FAM-CCG CCC TGG ACA CC-MGB.NFQ-3′	
HSV-2 forward	Before 2010	5′-CAA GCT CCC GCT AAG GAC AT-3′	Glycoprotein G, ORF 4 US fragment, 108 bp [19]
HSV-2 reverse	Before 2010	5′-GGT GCT GAT GAT AAA GAG GAT ATC TAG A-3′	
HSV-2 probe	Before 2010	5′-FAM-ACA CAT CCC CCT GTT CTG GTT CCT AAC G-TAMRA-3′	
VZV forward	Before 2010	5′-ACA GCT TGT CTT TAT TGG AGA GCA A-3′	Glycoprotein I, ORF 67 US fragment, 84 bp [16]
VZV reverse	Before 2010	5′-GCC ACC GTA TCC GCG TAT A-3′	
VZV probe	Before 2010	5′-FAM-ACC TAC CGG GAC AAA CTA TAG CGG AAC ACT G-TAMRA-3′	
	From 13 January 2025	5′-FAM-ACC TAC CGG GAC AAA CTA TAG CGG AAC ACT G-BHQ1-3′ (improving signal quality)	
GAPDH forward	Before 2010	5′-CAA GGT CAT CCA TGA CAA CTT TG-3′	Homo sapiens glyceraldehyde-3-phosphate dehydrogenase, 89 bp [16]
GAPDH reverse	Before 2010	5′-GGC CAT CCA CAG TCT TCT GG-3′	
GAPDH probe	Before 2010	5′-VIC-ACC ACA GTC CAT GCC ATC ACT GCC A-TAMRA-3′	
PhHV forward	From 28 August 2024	5′-GGG CGA ATC ACA GAT TGA ATC-3′	Phocid herpes virus (PhHV) glycoprotein B, 89 bp [15]
PhHV reverse	From 28 August 2024	5′-GCG GTT CCA AAC GTA CCA A-3′	
PhHV probe	From 28 August 2024	5′-VIC-TTT TTA TGT GTC CGC CAC CAT CTG GAT C-BHQ1-3′	

HSV-1: Herpes simplex virus type 1; HSV-2: Herpes simplex virus type 2; VZV: Varicella zoster-virus; CMV: cytomegalovirus; GAPDH: Homo sapiens glyceraldehyde-3-phosphate dehydrogenase; PhHV: phocid herpes virus.

**Table 2:** Total amount of PCR assays from corneal and conjunctival scrapings conducted between January 2010 and March 2025.

Virus	Assays Performed	Positives (*n*)	Positivity (%)	Median Age (Years)	IQR (25th to 75th Percentile)	Male (%)	Female (%)
HSV-1	3358	328	9.8	52.2	34.3–71.9	59.4	40.6
HSV-2	3290	9	0.27	57.2	46.7–66.4	22.2	77.8
VZV	3112	143	4.6	55.0	38.1–73.1	48.0	52.0
CMV	194	2	1.0	37.5	35.8–39.2	100	0

**Table 3:** Total amount of PCR assays from simultaneous HSV-1, HSV-2 and VZV “triplets”. The percentages in gender distribution refer to positive tested patients only.

Virus	Triplet Panels	Positive Patients (*n*)	Positivity (%)	Median Age (Years)	IQR (25th to 75th Percentile)	Male (%)	Female (%)
HSV-1	2913	274	9.4	55.3	38.2–73.3	58.9	41.1
HSV-2	2913	9	0.4	57.2	46.7–66.4	33.3	66.7
VZV	2913	136	6.2	53.7	37.0–72.1	47.9	52.1

**Table 4:** Annual number of triplet test panels and virus detections conducted from January 2010 until March 2025 at the university hospital of Zurich in Switzerland.

Year	Triplet Panels	HSV-1 Pos	HSV-2 Pos	VZV Pos	CMV Pos
2010	114	12	0	4	0
2011	99	14	1	16	1
2012	140	18	0	5	1
2013	210	21	0	7	0
2014	223	13	3	19	0
2015	218	24	1	23	0
2016	218	28	2	13	0
2017	238	20	0	3	0
2018	166	14	0	9	0
2019	200	24	0	5	0
2020	138	14	0	4	0
2021	173	17	1	4	0
2022	198	9	0	8	0
2023	234	15	0	7	0
2024	268	22	0	5	0
2025	76	9	1	4	0
Total	2913	274	9	136	2

Number of corneal- and conjunctival-scraping specimens for which HSV-1, HSV-2, and VZV PCRs were run simultaneously (“triplet panels”), together with the annual counts of PCR-positive specimens for HSV-1, HSV-2, VZV, and CMV.

## Data Availability

The original contributions presented in this study are included in the article. Further inquiries can be directed to the corresponding author. Since this is a non-public project data will be made available upon request to the correspondence author.

## Abbreviations

The following abbreviations are used in this manuscript:

HSV-1	Herpes simplex virus type 1
HSV-2	Herpes simplex virus type 2
VZV	Varicella zoster virus
CMV	Cytomegalovirus
PCR	Polymerase chain reaction
GAPDH	Homo sapiens glyceraldehyde-3-phosphate dehydrogenase
PhHV	Phocid herpes virus
